# Sales of anti-cancer medicines; China, Indonesia, Kazakhstan, Malaysia, Philippines and Thailand

**DOI:** 10.2471/BLT.19.243998

**Published:** 2020-05-28

**Authors:** Alessandra Ferrario, Peter Stephens, Xiaodong Guan, Dennis Ross-Degnan, Anita Wagner

**Affiliations:** a Harvard Medical School and Harvard Pilgrim Healthcare Institute, 401 Park Drive, Suite 401 East, Boston, MA 02215 United States of America.; b IQVIA, London, England.; c School of Pharmaceutical Sciences, Peking University, Beijing, China.

## Abstract

**Objective:**

To assess sales of anti-cancer medicines in the 2017 World Health Organization’s *WHO Model list of essential medicines* in China, Indonesia, Kazakhstan, Malaysia, Philippines and Thailand from 2007 (2008 for Kazakhstan and Malaysia) to 2017.

**Methods:**

We extracted sales volume data for 39 anti-cancer medicines from the IQVIA database. We divided the total quantity sold by the reference defined daily dose to estimate the total number of defined daily doses sold, per country per year, for three types of anti-cancer therapies (traditional chemotherapy, targeted therapy and endocrine therapy). We adjusted these data by the number of new cancer cases in each country for each year.

**Findings:**

We observed an increase in sales across all types of anti-cancer therapies in all countries. The largest number of defined daily doses of traditional chemotherapy per new cancer case was sold in Thailand; however, the largest relative increase per new cancer case occurred in Indonesia (9.48-fold). The largest absolute and relative increases in sales of defined daily doses of targeted therapies per new cancer case occurred in Kazakhstan. Malaysia sold the largest number of adjusted defined daily doses of endocrine therapies in 2017, while China and Indonesia more than doubled their adjusted sales volumes between 2007 and 2017.

**Conclusion:**

The use of sales data can fill an important knowledge gap in the use of anti-cancer medicines, particularly during periods of insurance coverage expansion. Combined with other data, sales volume data can help to monitor efforts to improve equitable access to essential medicines.

## Résumé

**Objectif:**

Évaluer la vente de médicaments contre le cancer figurant dans l'édition 2017 de la *Liste modèle des médicaments essentiels* publiée par l'Organisation mondiale de la Santé en Chine, en Indonésie, au Kazakhstan, en Malaisie, aux Philippines et en Thaïlande entre 2007 (2008 pour le Kazakhstan et la Malaisie) et 2017.

**Méthodes:**

Nous avons extrait de la base de données IQVIA les informations relatives au volume de vente pour 39 médicaments contre le cancer. Nous avons divisé le nombre total de médicaments vendus par la dose quotidienne déterminée de référence, afin d'estimer le nombre total de doses quotidiennes déterminées vendues par pays et par an, pour trois types de traitements contre le cancer (chimiothérapie conventionnelle, thérapie ciblée et endocrinothérapie). Nous avons ajusté ces données en tenant compte du nombre de nouveaux cas de cancer diagnostiqués chaque année dans chaque pays.

**Résultats:**

Nous avons observé une hausse des ventes pour tous les types de traitements contre le cancer dans tous les pays. C'est la Thaïlande qui vend le plus grand nombre de doses quotidiennes déterminées en chimiothérapie conventionnelle pour chaque nouveau cas de cancer; néanmoins, c'est en Indonésie que nous avons constaté la plus grande augmentation relative pour chaque nouveau cas de cancer (nombre multiplié par 9,48). En termes d'augmentation absolue et relative des ventes de doses quotidiennes déterminées pour lathérapie ciblée, c'est au Kazakhstan qu'elle était la plus élevée. La Malaisie est le pays ayant vendu le plus grand nombre de doses quotidiennes déterminées pour l'endocrinothérapie, tandis que la Chine et l'Indonésie ont plus que doublé leur volume de vente ajusté entre 2007 et 2017.

**Conclusion:**

L'exploitation des données de vente peut combler un manque de connaissances dans l'utilisation de médicaments contre le cancer, surtout pendant les périodes d'élargissement de la couverture maladie. En les associant à d'autres informations, les données sur le volume de vente permettent de suivre les efforts fournis pour garantir un accès de plus en plus équitable aux médicaments essentiels.

## Resumen

**Objetivo:**

Evaluar las ventas de los medicamentos contra el cáncer que aparecen en la *Lista modelo OMS de medicamentos esenciales* de 2017 de la Organización Mundial de la Salud en China, Filipinas, Indonesia, Kazajstán, Malasia y Tailandia desde 2007 (2008 en el caso de Kazajstán y Malasia) hasta 2017.

**Métodos:**

Los datos sobre el volumen de ventas de 39 medicamentos contra el cáncer se obtuvieron de la base de datos IQVIA. Se dividió la cantidad total que se vendió por la dosis diaria definida de referencia para estimar el número total de dosis diarias definidas que se vendieron, por país y por año, para tres tipos de tratamientos anticancerosos (quimioterapia tradicional, terapia dirigida y tratamiento endocrino). Se adaptaron estos datos por el número de nuevos casos de cáncer en cada país para cada año.

**Resultados:**

Se observó un incremento en las ventas de todos los tipos de tratamientos anticancerosos en todos los países. El mayor número de dosis diarias definidas de quimioterapia tradicional por cada nuevo caso de cáncer se vendió en Tailandia; sin embargo, el mayor incremento relativo por cada nuevo caso de cáncer se registró en Indonesia (9,48 veces). Los mayores incrementos absolutos y relativos de las ventas de dosis diarias definidas de las terapias dirigidas por cada nuevo caso de cáncer se registraron en Kazajstán. Malasia vendió el mayor número de dosis diarias definidas adaptadas de los tratamientos endocrinos en 2017, mientras que China e Indonesia duplicaron con creces sus volúmenes de ventas ajustadas entre 2007 y 2017.

**Conclusión:**

El empleo de los datos de ventas puede llenar un importante vacío de conocimientos sobre el uso de los medicamentos contra el cáncer, en particular durante los periodos de la ampliación de la cobertura de los seguros. Los datos sobre el volumen de ventas, junto con otros datos, pueden ayudar a supervisar los esfuerzos por mejorar el acceso equitativo a los medicamentos esenciales.

## ملخص

الغرض تقييم مبيعات أدوية مكافحة السرطان في القائمة النموذجية للأدوية الأساسية لمنظمة الصحة العالمية (WHO) لعام 2017 في كل من الصين وإندونيسيا وكازاخستان وماليزيا والفلبين وتايلند، من عام 2007 (2008 بالنسبة لكازاخستان وماليزيا) حتى عام 2017.

الطريقة قمنا باستخلاص بيانات حجم المبيعات لعدد 39 دواءً مكافحاً للسرطان من قاعدة بيانات IQVIA. قمنا بتقسيم إجمالي الكمية المباعة بالجرعة اليومية المرجعية المحددة، وذلك لتقدير إجمالي عدد الجرعات اليومية المحددة المباعة، لكل بلد سنوياً، لثلاثة أنواع من العلاجات المكافحة للسرطان (العلاج الكيميائي التقليدي، والعلاج الموجه، والعلاج بالغدد الصماء). قمنا بتعديل هذه البيانات حسب عدد حالات السرطان الجديدة في كل دولة لكل عام.

النتائج لاحظنا زيادة في مبيعات كل أنواع العلاجات المكافحة للسرطان في كل الدول. تم بيع أكبر عدد من الجرعات اليومية المحددة من العلاج الكيميائي التقليدي، لكل حالة سرطان جديدة، في تايلند؛ ومع ذلك، حدثت أكبر زيادة نسبية لكل حالة سرطان جديدة في إندونيسيا (9.48 ضعفاً). حدثت أكبر زيادات مطلقة ونسبية في مبيعات الجرعات اليومية المحددة من العلاجات المستهدفة لكل حالة سرطان جديدة، في كازاخستان. باعت ماليزيا أكبر عدد من الجرعات اليومية المعدلة من علاجات الغدد الصماء في عام 2017، بينما باعت الصين وإندونيسيا أكثر من ضعف حجم مبيعاتهما المعدلة بين عامي 2007 و2017.

الاستنتاج إن استخدام بيانات المبيعات يمكنه أن يسد فجوة معرفية هامة في استخدام أدوية مكافحة السرطان، وخاصة خلال فترات تمديد التغطية التأمينية. يمكن لبيانات حجم المبيعات، إلى جانب بيانات أخرى، أن تساعد في مراقبة الجهود لتحسين الوصول العادل إلى الأدوية الأساسية. ****

## 摘要

**目的:**

旨在评估世界卫生组织发布的*《世卫组织基本药物标准清单》*中的抗癌药物于 2007 年至 2017 年在菲律宾、哈萨克斯坦、马来西亚、泰国、印度尼西亚和中国（2008 年哈萨克斯坦和马来西亚）的销售情况。

**方法:**

我们从 IQVIA 数据库中选取了 39 种抗癌药物的销量数据。我们用销售总量除以此参考清单中的限定日剂量来估算每个国家三种抗癌疗法（传统化疗、靶向治疗和内分泌疗法）每年的限定日剂量总销量。我们根据每个国家每年新增癌症病例数调整了这些数据。

**结果:**

我们观察到所有国家各种类型的抗癌疗法的销售额都有所增长。泰国每例新增癌症患者购买传统化疗药物的限定日剂量最大；然而，印度尼西亚每例新增癌症患者的相对增幅最大（9.48 倍）。哈萨克斯坦的新增癌症患者中，靶向治疗的限定日剂量销售额呈现最大的绝对增幅和相对增幅。2017 年，马来西亚调整后的内分泌疗法限定日剂量销量最大，而中国和印度尼西亚的调整后销售量在 2007 年至 2017 年增加了一倍以上。

**结论:**

销售数据的使用可以填补抗癌药物使用方面的重要认知缺口，尤其是在扩大保险覆盖范围期间。结合其他数据，销量数据有助于监测改善患者平等获取基本药物方面的工作。

## Резюме

**Цель:**

Оценить уровень продаж противораковых препаратов из изданного Всемирной организацией здравоохранения в 2017 году*****Примерного перечня основных лекарственных средств*****в Индонезии, Казахстане, Китае, Малайзии, Таиланде и на Филиппинах в период с 2007 года (с 2008 года для Казахстана и Малайзии) по 2017 год.

**Методы:**

Авторы получили данные о продажах 39 противораковых препаратов из базы данных IQVIA. Общее количество проданных единиц было разделено на эталонную условную суточную дозу для оценки общего количества условных суточных доз, проданных в конкретной стране за год. Рассматривались три типа противораковых препаратов: традиционная химиотерапия, таргетная и гормональная терапия. Данные затем были скорректированы по количеству новых случаев рака в каждой стране за каждый год.

**Результаты:**

Наблюдался рост продаж по всем типам противораковых препаратов в каждой из стран. Максимальное количество условных суточных доз традиционной химиотерапии на новый случай рака было продано в Таиланде, однако максимальный относительный прирост по каждому новому случаю продемонстрировала Индонезия (рост в 9,48 раза). Максимальный абсолютный и относительный прирост продаж условных суточных доз препаратов для таргетной терапии на новый случай рака продемонстрировал Казахстан. Малайзия стала лидером по продажам скорректированных условных суточных доз препаратов для гормональной терапии в 2017 году, а в период с 2007 по 2017 год Индонезия и Китай увеличили скорректированные объемы продаж более чем вдвое.

**Вывод:**

Использование данных продаж поможет восполнить значительный пробел в знаниях о применении противораковых препаратов, в частности в периоды расширения страхового покрытия. В сочетании с другими данными показатели объемов продаж помогут отслеживать усилия по обеспечению равного доступа населения к основным лекарственным средствам.

## Introduction

The sustainable development goals (SDG) identify access to quality medicines as a key component of universal health coverage (UHC).^1^ The SDGs also reflect a global recognition of the need to tackle the growing burden of noncommunicable diseases, including cancer. The World Health Organization’s* WHO Model list of essential medicines* includes a section on anti-neoplastic medicines that countries can use to inform the development of their own national essential medicines lists. Beyond inclusion in national essential medicines lists,^2^^–^^6^ little is known about the use of cancer medicines at a country level. This is an important gap, particularly as countries are striving to improve access to care and financial protection through UHC.

The Lancet Commission on Essential Medicines for Universal Health Coverage^7^^,^^8^ called for the continuous and global monitoring of access to essential medicines (including anti-cancer medicines) in the form of routine data disaggregated by gender, ethnicity, education, residential location and wealth quintile. As these data are not readily available in many settings, it is important to leverage different sources of evidence to inform progress.^9^^,^^10^ Multicountry studies on access to anti-cancer medicines in low- and middle-income countries have used the inclusion of medicines in national essential medicines or reimbursement lists and formularies^2^^–^^6^^,^^11^ and their availability on the market and applicable copayments^11^ as proximate indicators of access. Country-level studies have used national essential medicines lists and formularies,^12^^,^^13^ sales data,^14^^–^^16^ surveys^17^ and patient-level data from medical records^18^^–^^23^ to assess access to anti-cancer medicines in low- and middle-income countries. However, to our knowledge, few of the existing studies^15^ or official statistics^24^ have assessed the changes in use of anti-cancer medicines during the past decade of coverage expansion in low- and middle-income countries.

As the number of new cancer cases worldwide is expected to increase from 18.1 million in 2018 to 29.5 million in 2040^25^ and the need to improve access to medicines is high on the global agenda,^8^ we need evidence on the use of anti-neoplastic medicines at a country level. In this study, we use routinely collected market sales data to assess how sales of the anti-cancer medicines included in the 2017 *WHO Model list of essential medicines*^26^ have evolved over time in six countries.

## Methods

### Country selection

We selected countries to include in our study based on the following criteria. First, we identified countries working towards UHC, and that have set up national public or private third-party payment systems or implemented other relevant pharmaceutical coverage policies within the government health system, in the last decade. Second, we chose countries in which IQVIA sales data on the medicines of interest were available and cover both public and private sectors, and hospital, as well as retail sectors. Third, we focused on countries from the same income group (i.e. middle income) and continent to make comparisons more meaningful. China, Indonesia, Kazakhstan, Malaysia, Philippines and Thailand met these criteria. We provide a summary of the demographic and socioeconomic characteristics of these six countries in Table 1.

**Table 1:** Demographic and socioeconomic characteristics of the six countries included in a study of anti-cancer medicine sales during 2007–2017

Characteristic	China	Indonesia	Kazakhstan	Malaysia	Philippines	Thailand
Population in 2017 (million)^a^	1386.4	264.6	18.0	31.1	105.2	69.2
Percentage of the population aged 0–14 years in 2017^b^	17.9	26.9	27.9	24.3	31.5	17.4
Percentage of the population aged ≥ 65 years in 2017^c^	10.3	5.7	7.1	6.4	4.9	11.4
GDP per capita, PPP in 2017 (current international dollars)^d^	16 782.2	12 279.2	26 490.8	30 025.2	8340.3	17 917.2
Current health expenditure per capita, PPP in 2017 (current international dollars)^e^	841.1	367.9	820.4	1110.4	371.7	670.9
Domestic general government health expenditure in 2017 (% of total health expenditure)^e^	56.7	49.1	62.2	52.0	35.0	79.0
All-cancer incidence per 100 000 population in 2017^f^	325	120	215	155	122	224

GDP: gross domestic product; PPP: purchasing power parity.

^a^ The World Bank.^27^

^b^ The World Bank.^28^

^c^ The World Bank.^29^

^d^ The World Bank.^30^

^e^ WHO Global Health Expenditure Database.^31^

^f^ Global Burden of Disease Collaborative Network.^32^ Compare data with the global all-cancer incidence per 100 000 population of 319 in 2017.^32^

### Medicine selection

We considered all 49 anti-neoplastic medicines (defined on the basis of their active ingredients) listed in sections 8.2 “Cytotoxic and adjuvant medicines” and 8.3 “Hormones and antihormones” of the 2017 WHO model list.^26^ We excluded five supportive medications used to prevent or relieve the side-effects of anti-neoplastic treatment. We also excluded five medications that are listed in other therapeutic classes, as well as in sections 8.2 or 8.3. For example, methotrexate is also listed in section 30.2 of the model list for the treatment of rheumatoid arthritis; based on the formulation of methotrexate, it is not possible to distinguish between indications (further details available in the data repository).^33^ The number of medicines that we selected for this study was therefore 39 (Box 1).

Box 1 **Anti-neoplastic drugs and their defined daily dose^a^ included in the study of anti-cancer medicine sales in six countries, 2007–2017**

**Traditional chemotherapy**

Asparaginase 14 000 units; Bendamustine 17 mg; Bleomycin 3 mg; Capecitabine 3000 mg; Carboplatin 25 mg; Chlorambucil 2 mg;^b^ Cisplatin 6.75 mg; Cyclophosphamide 250 mg; Cytarabine 50 mg;^b^ Dacarbazine 100 mg; Dactinomycin 0.32 mg; Daunorubicin 20 mg;^b^ Docetaxel 6.43 mg; Doxorubicin 5 mg; Etoposide 50 mg; Fludarabine 10 mg;^b^ Fluorouracil 150 mg; Gemcitabine 200 mg; Hydroxycarbamide 1750 mg; Ifosfamide 700 mg; Irinotecan 30 mg; Mercaptopurine 175 mg; Oxaliplatin 11 mg; Paclitaxel 15 mg; Procarbazine 50 mg;^b^ Tioguanine 25 mg;^b^ Tretinoin (or all-trans retinoid acid) 10 mg;^b^ Vinblastine 11.61 mg; Vincristine 360 mg; and Vinorelbine 18 mg

**Targeted therapies**

Dasatinib 120 mg; Imatinib 500 mg; Nilotinib 600 mg; Rituximab 32 mg; and Trastuzumab 20 mg

**Endocrine therapy**

Anastrozole 1 mg; Bicalutamide 50 mg; Leuprorelin 1 mg; and Tamoxifen 20 mg

^a^ Defined daily dose according to German Institute for Medical Documentation,^34^ unless otherwise indicated.^b^ Defined daily dose were not available from the German Institute for Medical Documentation, we therefore adopted the strength of the smallest common unit (e.g. smallest tablet) as defined daily dose.

### Data source

IQVIA conducts multisample audits of pharmaceutical purchase data based on invoices from pharmacies, wholesalers, distributors and manufacturers in the hospital and retail sectors in several countries worldwide (further details available in data repository).^33^^,^^35^^,^^36^ The proprietary data are extrapolated to represent national-level sales. Quality checks (e.g. comparison with manufacturer data) are conducted to ensure the accuracy and representativeness of the data.^36^ We extracted quarterly data on sales volumes of all anti-neoplastic medicines of interest from 2007 to 2017 from the IQVIA database; complete data for Kazakhstan and Malaysia were only available from 2008 onwards, so we used 2008 as the baseline for both these countries. The data set included country, setting (retail or hospital), generic name, quarter, year, strength, formulation, units per pack and number of packs sold.

The assumed average maintenance dose per day for a medicine used for its main indication in adults, defined by the Anatomical Therapeutic Chemical Classification System according to its administration route, is referred to as the defined daily dose.^37^ The defined dose provides a common unit of analysis that can be used to study the use of medicines over time and across therapeutic classes and population groups. The WHO Collaborating Centre for Drug Statistics Methodology assigns a defined daily dose index for many drugs, but made the decision not to assign defined daily doses for medicines with highly individualized treatment schedules, such as anti-neoplastic medicines.^37^ We therefore used defined daily dose information provided by the German Institute for Medical Documentation and Information, which defines daily dose based on the standard dose in the manufacturer submission for marketing authorization.^34^ If we were not able to obtain a defined daily dose for a particular medicine from the German Institute for Medical Documentation and Information, we adopted the smallest common unit used in the six countries as a reference (Box 1).

We used country-specific estimates of new cancer cases available by year for the entire study period from the 2017 Global Burden of Disease (GBD) study.^32^

### Analysis

Using information on the strength of each medicine, number of units per pack and number of packs sold, we estimated the total milligrams (or active units for asparaginase) sold. We divided the total quantity sold by the reference defined daily dose for that particular medicine to estimate the total number of defined daily doses sold. We defined sales volume as the estimated number of defined daily doses sold per country and per year for each of the three types of anti-cancer treatment (traditional chemotherapy, targeted therapy and endocrine therapy). We also calculated the number of defined daily doses sold per new cancer case.

### Ethics

Ethical approval was granted by the Institutional Review Board of Harvard Pilgrim Health Care.

## Results

### Cancer incidence

Of the countries studied, China is the most populous and also, according to GBD data,^32^ the country with the highest all-cancer incidence (325 per 100 000 in 2017; Table 1). The highest number of new cancer cases for both 2007 and 2017 was observed in China, as well as the largest absolute (1 807 036.83 cases) and relative (1.65-fold) increase (Table 2). GBD data^32^ indicate that Indonesia, the second-most populous of the countries studied, had the second-highest number of cancer cases in both 2007 and 2017 and the second-largest increase in absolute numbers (68 505.81 cases), but not in relative numbers (1.28-fold). We noted that the second-highest relative increase in the number of new cancer cases (1.53-fold; from 2008 to 2017 in this case) occurred in Malaysia.

**Table 2:** All-cancer incidence and sales volumes of anti-cancer medicines in six countries in 2007 (or 2008) and 2017

Data	China	Indonesia	Kazakhstan^a^	Malaysia^a^	Philippines	Thailand
**New cancer cases, all cancers^b^**						
Year 2007 or 2008^a^	2 781 760.17	242 435.72	35 281.97	31 045.63	87 922.57	120 420.79
Year 2017	4 588 797.00	310 941.53	38 503.55	47 594.47	126 203.23	157 914.48
Absolute increase	1 807 036.83	68 505.81	3221.57	16 548.84	38 280.66	37 493.69
Relative increase, *x*-fold)	1.65	1.28	1.09	1.53	1.44	1.31
**Chemotherapy**						
Total no. defined daily doses sold^c^						
Year 2007 or 2008^a^	80 222 871.95	965 421.50	1 075 377.68	2 023 814.87	1 594 213.68	6 182 965.79
Year 2017	202 691 916.50	11 737 244.72	1 307 816.88	2 715 687.49	3 867 712.03	12 354 259.40
Absolute increase	122 469 044.55	10 771 823.22	232 439.20	691 872.62	2 273 498.34	6 171 293.91
Relative increase, *x*-fold	2.53	12.16	1.22	1.34	2.43	2.00
No. defined daily doses sold per new cancer case						
Year 2007 or 2008^a^	28.84	3.98	30.48	65.19	18.13	51.34
Year 2017	44.17	37.75	33.97	57.06	30.65	78.23
Absolute increase	15.33	33.77	3.49	−8.13	12.51	26.89
Relative increase, *x*-fold	1.53	9.48	1.11	0.88	1.69	1.52
**Targeted therapy**						
Total no. defined daily doses sold^c^						
Year 2007 or 2008^a^	333 010.55	9 685.43	1 253.75	39 124.13	37 116.25	125 958.25
Year 2017	11 281 751.09	488 889.37	365 575.96	400 541.29	217 806.95	1 104 714.19
Absolute increase	10 948 740.54	479 203.94	364 322.21	361 417.17	180 690.70	978 755.94
Relative increase, *x*-fold	33.88	50.48	291.59	10.24	5.87	8.77
No. defined daily doses sold per new cancer case						
Year 2007 or 2008^a^	0.12	0.04	0.04	1.26	0.42	1.05
Year 2017	2.46	1.57	9.49	8.42	1.73	7.00
Absolute increase	2.34	1.53	9.46	7.16	1.31	5.95
Relative increase, *x*-fold	20.54	39.36	267.19	6.68	4.09	6.69
**Endocrine therapy**						
Total no. defined daily doses sold^c^						
Year 2007 or 2008^a^	34 219 204.50	1 394 979.75	1 771 683.00	3 066 676.50	2 890 588.06	6 003 971.15
Year 2017	129 391 344.55	3 979 765.31	2 323 521.75	5 338 724.85	5 955 772.99	11 275 412.09
Absolute increase	95 172 140.05	2 584 785.56	551 838.75	2 272 048.35	3 065 184.93	5 271 440.94
Relative increase, *x*-fold	3. 78	2.85	1.31	1.74	2.06	1.88
No. defined daily doses sold per new cancer case						
Year 2007 or 2008^a^	12.30	5.75	50.21	98.78	32.88	49.86
Year 2017	28.20	12.80	60.35	112.17	47.19	71.40
Absolute increase	15.90	7.05	10.13	13.39	14.32	21.54
Relative increase, *x*-fold	2.29	2.22	1.20	1.14	1.44	1.43

^a^ Complete sales data for Kazakhstan and Malaysia were only available from 2008, therefore we used the 2008 data as the baseline for these countries.

^b^ Global Burden of Disease Collaborative Network.^32^

^c^ Data available on request from IQVIA.

### Sales volumes

The sales data indicated that, of the six countries studied, the highest number of defined daily doses of traditional chemotherapy was sold in China in both 2007 and 2017 (Table 2); China also demonstrated the highest absolute increase in defined daily doses of traditional chemotherapy sold from 2007 to 2017. However, the largest relative increase (12.16-fold) was observed in Indonesia.

**Fig. 1:**
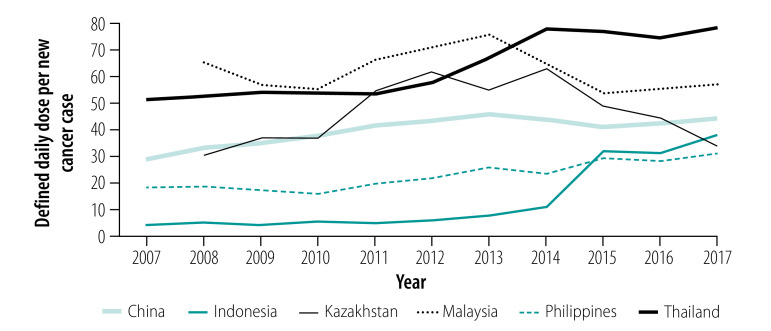
Defined daily doses of traditional chemotherapy sold per new cancer case in six countries from 2007 (or 2008) to 2017

Note: Data for Kazakhstan and Malaysia were only available from 2008, therefore we used the 2008 data as the baseline for these countries.

Source: IQVIA and Global Burden of Disease Collaborative Network.^32^

**Fig. 2:**
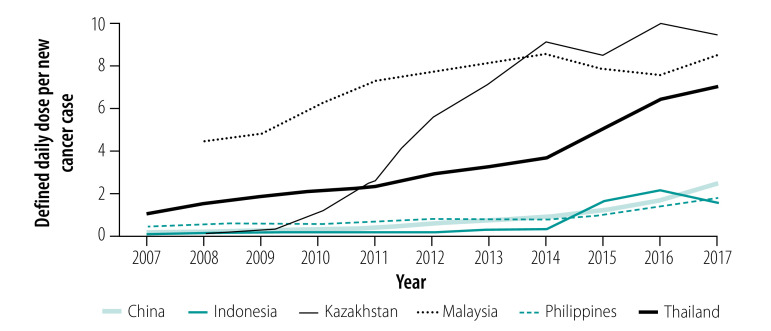
Defined daily doses of targeted therapy sold per new cancer case in six countries from 2007 (or 2008) to 2017

Note: Data for Kazakhstan and Malaysia were only available from 2008, therefore we used the 2008 data as the baseline for these countries.

Source: IQVIA and Global Burden of Disease Collaborative Network.^32^

**Fig. 3:**
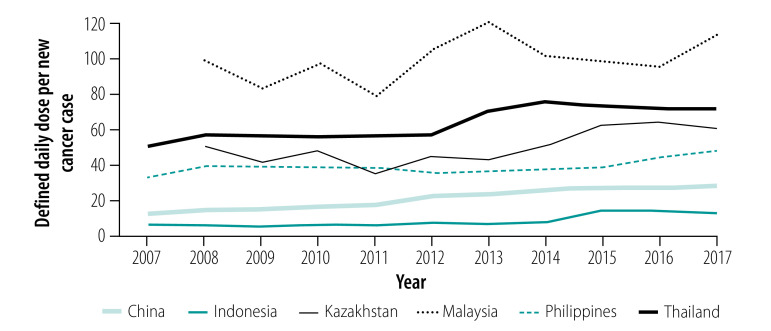
Defined daily doses of endocrine therapy sold per new cancer case in six countries from 2007 (or 2008) to 2017

Note: Data for Kazakhstan and Malaysia were only available from 2008, therefore we used the 2008 data as the baseline for these countries.

Source: IQVIA and Global Burden of Disease Collaborative Network.^32^

Overall, the number of defined daily doses of traditional chemotherapy, targeted therapy and endocrine therapy sold per new cancer case increased over time for all six countries studied, with the exception of traditional chemotherapy in Malaysia (Fig. 1, Fig. 2 and Fig. 3).

In terms of defined daily doses per incident cancer case, for traditional chemotherapy the greatest relative increase from baseline occurred in Indonesia, where sales increased from 3.98 in 2007 to 37.75 in 2017. The highest number of defined daily doses per new case in 2017 was sold in Thailand (78.23). Although the total sales volume (in defined daily doses) increased in Malaysia from 2008 (2 023 814.87) to 2017 (2 715 687.49), this increase was by a lower proportion (1.34-fold) than the reported increase in the number of new cancer cases (1.53-fold); Malaysia therefore observed a decrease from 65.19 defined daily doses sold per new case in 2008 to 57.06 in 2017. 

For targeted therapies, the highest number of defined daily doses per new cancer case in 2017 was sold in Kazakhstan, in which we observed the largest relative increase from 0.04 in 2008 to 9.49 in 2017. Of the five targeted therapies considered in this study (Box 1), trastuzumab and imatinib were sold in the largest quantities per new cancer case in all countries. For endocrine therapies, the largest numbers of defined daily doses of endocrine therapies per new cancer case (112.17) were sold in Malaysia in 2017, while both China and Indonesia more than doubled their numbers of defined daily doses sold per new cancer case from 2007 to 2017. Of the four endocrine medicines included in this study (Box 1), tamoxifen was the most widely sold endocrine therapy in all countries.

## Discussion

Our data show an overall increase in the sales of anti-cancer therapies in China, Indonesia, Kazakhstan, Malaysia (increase in overall chemotherapy sold, although a slight decrease per new cancer case), Philippines and Thailand during our study period. During this time, the six countries continued working towards the goals of achieving UHC. Given the high cost of cancer treatment, the expansions these countries have made in health-care coverage over the last decade will likely have played an important role in enabling greater use of these medicines. The largest increase occurred in defined daily doses of traditional chemotherapy sold in Indonesia between 2014 and 2015, which is when implementation of UHC began in that country. In addition to a higher availability of cancer medicines, improved access to a health-care system as a result of progress in UHC could have led to earlier diagnoses (at a stage amenable to treatment), contributing to an increased use of cancer medicines.

While the overall increase in sales of anti-cancer medicines from 2007 (or 2008) to 2017 in each country suggests increased access by the populations, aggregated results hide potential disparities in access between groups within a population. Two studies in Thailand found differences in the use of medicines between members of different insurance schemes. One study described differences in the type of treatment received and health outcomes between individuals with colorectal cancer insured under the UHC scheme for the general population and those in the civil servant scheme.^22^ Disparities in survival were also found for lymphoma patients, and mainly attributed to limited access to rituximab for UHC-insured patients at the time of the study (2003–2006).^23^ Two studies from China on patterns of prescribing for patients with breast cancer highlighted limited access to trastuzumab for patients overexpressing human epidermal growth factor receptor 2 (HER-2).^18^^,^^20^ One of these two multicentre studies found that, between 2011 and 2014, only 31 (28.4%) of 109 patients overexpressing HER-2 were treated with trastuzumab.^20^ A 2011 study in Malaysia found that only 19% of 172 patients younger than 70 years with HER-2-positive breast cancer stage I to III received trastuzumab within 1 year of diagnosis, which the authors attributed to its high cost and insufficient public funding for the treatment.^21^ Despite these earlier findings highlighting limited access, official statistics show that the use of trastuzumab has increased over time (particularly in the public sector) following inclusion in the national formulary in 2008.^24^

As global spending on cancer treatment continues to rise, new therapies continue to enter the market and new indications are being approved for medicines already available,^38^ countries will increasingly face challenges in enabling access to new therapies in an equitable way. Budgetary constraints play an important role in access to highly priced medicines, such as the five targeted therapies included in this study. In the absence of large discounts as part of differential pricing or industry access programmes, it will be challenging for middle-income countries working towards UHC to provide cancer medicines equitably to all patients in need. In Thailand for example, UHC-insured patients have access to medicines on the national essential medicines list and civil servants have access to all medicines on the Thai market.^14^^,^^15^ To facilitate the coverage of highly priced medicines, Thailand has engaged in compulsory licensing, price negotiation and health technology assessment, and created a separate section on the essential medicines list (E2) used for centralized procurement, among other measures.^15^ Similarly, in China, coverage of medicines differed between provinces and insurance schemes before 2018.^39^ Generally, coverage with the Urban Employee Basic Medical Insurance scheme is more generous than the Urban Resident Basic Medical Insurance scheme and both provide greater coverage than the New Rural Cooperative Medical Scheme. To help municipalities (ultimately responsible for payment of medicines) cover medicines in the Basic Medical Insurance scheme, the Chinese Government has engaged in price negotiation with manufacturers to secure more competitive prices. Further, since 2019, provinces are required to cover all medicines included in the Basic Medical Insurance list (including the national essential medicines list); previously there was some flexibility for provinces to either exclude some medicines listed as essential (in the national list) from coverage, or to cover some medicines not listed as essential.^40^

Our study had several limitations. First, we used national-level sales data of anti-cancer medicines included in the 2017 *WHO Model list of essential medicines*; sales data do not tell us whether the medicine was eventually prescribed, dispensed and administered to the patient. If procurement quantities were inaccurate, or less patients presented than in previous years, sales may differ from actual use. Anti-cancer treatments included in the 2017 WHO model list are a subset of all available anti-cancer medicines; this subset is selected by the WHO Expert Committee on the Selection and Use of Essential Medicines by considering the burden of disease and responsiveness of the indicated cancer to pharmacotherapy. We had no access to patient-level data on the actual conditions treated, regimens, insurance status, co-payments, income, education or the many other factors that can influence the use of medicines. Our measure, defined daily dose, is a way to summarize and compare sales volumes across products and does not necessarily reflect the way in which these medicines are actually prescribed. Since we had no information on the indications for which these medicines were used in clinical practice (and most of these medicines can be used for different types of cancers), we could only attempt a crude adjustment for use by the total number of new cancer cases per country and year. Furthermore, the quality of the available data on cancer incidence may differ between countries, which may impact the reliability of inter-country comparisons.

Second, we cannot judge whether the sales levels we observed are sufficient to appropriately treat all cancer patients in a given country. The sales differences we identified between the six countries cannot be attributed to any specific cause, such as financial barriers, training and infrastructure, lack of insurance coverage, differences in clinical guidelines or stage at diagnosis, all of which might partially explain sales differences.

Finally, while IQVIA data are nationally representative and include the relevant channels (public and private sectors; hospital and retail settings) where cancer medicines are transacted, medicines procured through special channels (e.g. donations) may not be included in the IQVIA sample and therefore may not be captured in the data.

Despite the limitations described here, studies using routinely aggregated sales data over time can fill an important knowledge gap in the use of medicines. Such studies can be complemented by other research based on higher-resolution patient-level data (e.g. health insurance claims data, medical records and socioeconomic status),^9^^,^^41^ allowing assessments of equity in access. Future studies also need to assess the affordability of cancer medicines at the household and system level. Monitoring sales in the context of insurance coverage expansions is important to identify progress and challenges in improving access to anti-cancer medicines, contributing to the SDG agenda. Our study has taken a first step in that direction.
